# Compression Molded Soy Protein Films with Exopolysaccharides Produced by Cider Lactic Acid Bacteria

**DOI:** 10.3390/polym12092106

**Published:** 2020-09-16

**Authors:** Jone Uranga, Mª Goretti Llamas, Ziortza Agirrezabala, María Teresa Dueñas, Oier Etxebeste, Pedro Guerrero, Koro de la Caba

**Affiliations:** 1 BIOMAT Research Group, University of the Basque Country (UPV/EHU), Escuela de Ingeniería de Gipuzkoa, Plaza de Europa 1, 20018 Donostia-San Sebastián, Spain; jone.uranga@ehu.eus; 2 GLYCOBAL Research Group, Facultad de Química, University of the Basque Country (UPV/EHU), Paseo Manuel de Lardizabal 3, 20018 Donostia-San Sebastián, Spain; goretti.llamas@ehu.es (M.G.L.); zagirrezabala001@ikasle.ehu.eus (Z.A.); mariateresa.duenas@ehu.eus (M.T.D.); oier.echeveste@ehu.eus (O.E.)

**Keywords:** soy protein isolate, exopolysaccharides, compression molded films

## Abstract

Two exopolysaccharide (EPS)-producing lactic acid bacteria (LAB) strains, *Liquorilactobacillus (L.) sp* CUPV281 and *Liquorilactobacillus (L.) mali* CUPV271, were isolated from Spanish apple must. Each of the strains produced a dextran, with different branching degrees, to be incorporated into soy protein isolate (SPI) film-forming formulations. Films were prepared by compression molding, a more rapid processing method than solution casting and, thus, with a greater potential for scaling-up production. Thermal analysis showed that SPI and EPS start the degradation process at temperatures above 190 °C, confirming that the compression temperature selected (120 °C) was well below the corresponding degradation temperatures. Resulting films were transparent and homogeneous, as shown by UV-Vis spectroscopy and SEM, indicating the good compatibility between SPI and EPS. Furthermore, FTIR analysis showed that the interactions between SPI and EPS were physical interactions, probably by hydrogen bonding among the polar groups of SPI and EPS. Regarding antifungal/fungistatic activity, LAB strains used in this study showed an inhibitory effect on germination of fungal spores.

## 1. Introduction

Exopolysaccharides (EPS) are polysaccharides secreted by bacteria into the extracellular matrix, or they can also remain loosely associated to the cell surface by electrostatic interactions. Among EPS, homopolysaccharides, containing only one type of monomer, or heteropolysaccharides, with two or more different monosaccharides, can be found [1]. These biodegradable polymers have high molecular weights and relevant physiological functions [2]. Their large variation in composition, charge and structure confers multiple applications [3]. Some EPS, such as dextrans, produced by lactic acid bacteria, are considered as generally recognized as safe (GRAS) substances as defined by the US Food and Drug Administration (FDA) [4] and constitute a new generation of natural, harmless and prebiotic additives [5]. In the food industry, these biopolymers are employed for thickening, gelling, emulsification, stabilization, crystallization prevention, fat replacement, encapsulation, dough rheology and food texture improvement [6,7,8,9,10]. Beside food industry, they are also used as cosmeceuticals, herbicides and insecticides [11,12].

In recent years, the demand for natural polymers has increased and, thus, the production of EPS from new sources has gained more interest [13]. Moreover, the properties of EPS can be modified by blending with other polymers. Indeed, polysaccharide–protein mixtures have been widely employed for both films and biomaterials production [14]. In particular, soy protein has received much attention since it is widely available at a relatively low cost and shows film-forming ability, biocompatibility, biodegradability, and excellent oxygen and oil barrier properties [15,16,17]. Soy protein is mainly constituted of 7S and 11S protein fractions; moreover, disulfide linkages are present due to sulfhydryl groups of 11S fraction [18]. In this context, the objectives of this work were to develop films using vegetal-derived raw materials, specifically soy protein and two EPS produced by lactic acid bacteria (LAB) strains from our collection, and assess the effect of those EPS in the properties of soy protein films.

## 2. Materials and Methods 

### 2.1. Materials

Soy protein isolate (SPI), PROFAM 974, was supplied by ADM Protein Specialities Division (Amsterdam, Netherlands). According to the information provided by the supplier, SPI has a minimum of 90% protein content on a dry basis, a maximum of 5% moisture, 4% fat, 5% ash, and its isoelectric point is 4.6. Glycerol with a purity of 99.01% was obtained from Panreac (Barcelona, Spain). 

The two EPS employed in this work were produced using two strains of lactic bacteria isolated from apple must, known as *Liquorilactobacillus* sp. CUPV281 (AST1), and *Liquorilactobacillus mali* CUPV271 (E11). The monosaccharide composition analysis determined glucose as the only monosaccharide present in both EPS. These EPS are homopolysaccharides, specifically dextrans, composed of a linear backbone of α-(1→6)-linked glucosidic residues with variable degrees of strain-specific α-(1→3) and less frequent α-(1→4) branching.

### 2.2. Bacterial and Fungal Strains and Their Growth Conditions

For EPS production, *L. mali* CUPV271 (E11) and *L.* sp. CUPV281 (AST1) were activated in MRS [19] medium supplemented with 2% sucrose instead of glucose. Then, the preculture was grown in a semidefined (SMD) medium supplemented with 2% sucrose and the final fermentation took place in 1 L of the same medium for 20 h at 28 °C in microaerophilic conditions (5% CO_2_). For the recovery of EPS, a previously described protocol was used [20]. Dextran yield was 4.20 ± 0.09 g/L for AST1 and 11.65 ± 1.15 g/L for E11.

For bacterial/fungal co-cultures, strain BD185 of *Aspergillus nidulans* (genotype: *pyrG89; argB2;* Δ*nkuA::argB, gfp::flbB, pyroA4; veA1*) [21,22] and LAB strains AC1 (used as a reference) [20], AST1 and E11 were co-cultured in MRS medium adjusted to pH 6.5, supplemented with glucose or sucrose as repressor or inducer of EPS production [20], and also the nutrients associated with the genetic markers of the fungal strain (pyridoxine; 0.05 µg/mL of culture medium; uracil: 0.55 mg/mL of culture medium; uridine: 1.2 mg/mL of culture medium). Asexual spore production in strain BD185 is inhibited when grown on this culture medium (see below). This does not occur under standard growth conditions in *Aspergillus* minimal medium (AMM) [23].

### 2.3. Preparation of Films

Films were prepared by compression molding. First, 7 g of SPI, 40 wt % water, 30 wt % glycerol and 0 or 5 wt % EPS were mixed (percentages on SPI dry basis). Then, 1.2 g of the mixture was thermally compacted using a caver laboratory press (Specac, Barcelona, Spain), previously heated up to 120 °C for 1 min, by applying a pressure of 4 MPa for 2 min. Films without EPS were designated as control films, while those prepared with the EPS produced by AST1 and E11 were named SPI-AST1 and SPI-E11, respectively. All films were conditioned in a controlled environment chamber at 25 °C and 50% relative humidity before testing.

### 2.4. Characterization of Films

#### 2.4.1. Color Measurement

Color parameters (L*, a*, b*) were determined using a CR-400 Minolta Chroma-Meter colorimeter (Konica Minolta, Valencia, Spain). Films were placed on the surface of a white standard plate (calibration plate values: L* = 97.39, a* = 0.03 and b* = 1.77) and color parameters were measured using the CIELAB color scale: L* = 0 (black) to L* = 100 (white), −a* (greenness) to +a* (redness), and −b* (blueness) to +b* (yellowness). Color difference (ΔE*) was calculated relative to the control film:

(1) $$\Delta E^{*}=\sqrt{{\left(\Delta L^{*}\right)}^{2}+\;{\left(\Delta a^{*}\right)}^{2}+\;{\left(\Delta b^{*}\right)}^{2}}$$

#### 2.4.2. Gloss Measurements

Film gloss was determined using a Multi Gloss 268 Plus gloss meter (Konica Minolta, Valencia, Spain). According to ASTM D523-14 [24], gloss values were measured at an incidence angle of 60°.

#### 2.4.3. Light Absorption

Light absorption was measured in the UV-Vis range (200–800 nm) using a V-630 UV-Vis spectrophotometer (Jasco, Madrid, Spain).

#### 2.4.4. Differential Scanning Calorimetry (DSC)

DSC was carried out in a Mettler Toledo DSC 822 device (Mettler Toledo S.A.E., Madrid, Spain). Samples (3.0 ± 0.2 mg) were sealed in aluminum pans to avoid mass loss during the experiment. Filled pans were heated from −50 to 250 °C at a rate of 10 °C/min under inert atmosphere conditions (10 mL N_2_/min) to avoid thermo-oxidative reactions.

#### 2.4.5. Thermo-Gravimetric Analysis (TGA)

Thermal stability was analyzed by TGA and measurements were performed in a Mettler Toledo TGA SDTA 851 equipment (Mettler Toledo S.A.E., Madrid, Spain). The samples were heated from 25 to 800 °C at a heating rate of 10 °C/min under inert atmosphere conditions (10 mL N_2_/min) to avoid thermo-oxidative reactions.

#### 2.4.6. Fourier Transform Infrared (FTIR) Spectroscopy

FTIR spectra of pure EPS and films were carried out on a Nicolet Nexus 380 FTIR spectrometer using ATR Golden Gate (Specac, Barcelona, Spain). A total of 32 scans were performed at a resolution of 4 cm^−1^ in the wavenumber range from 800 to 4000 cm^−1^.

#### 2.4.7. Moisture Content (MC)

To determine MC of films, specimens were weighed (*w*_0_) and then dried in an oven at 105 ºC for 24 h. After this time, samples were reweighed (*w*_1_) to determine their MC according to the following formula:

(2) $$\mathrm{MC}\;\left(\%\right)=\frac{w_{0}-w_{1}}{w_{0}}\cdot 100$$

#### 2.4.8. Buffer Uptake (BU)

In order to study the buffer uptake of films, first, film pieces were weighed (*w*_0_) and then, immersed into 20 mL phosphate buffer solutions (PBS, pH = 7.4) to simulate body fluids. Samples were weighed after immersion into buffer solution for specific times (*w*_t_), until constant values were obtained. BU was calculated according to the following equation:

(3) $$\mathrm{BU}\;\left(\%\right)=\frac{w_{t}-w_{0}}{w_{0}}\cdot 100$$

#### 2.4.9. X-ray Diffraction (XRD)

XRD analysis was performed with a diffraction unit (PANalytic Xpert PRO, Malvern Instruments, Malvern, Spain) operating at 40 kV and 40 mA. The radiation was generated from a Cu-Kα (λ = 1.5418 Å) source. The diffraction data were obtained from 2θ values from 2° to 50°, where θ is the incidence angle of the X-ray beam on the sample.

#### 2.4.10. Scanning Electron Microscopy (SEM)

The film inner morphology was visualized using a Hitachi S-4800 field emission scanning electron microscope (Hitachi High-Technologies Corporation, Madrid, Spain). Samples were mounted on a metal stub with a double-side adhesive tape and coated under vacuum with gold (JFC-1100) in an argon atmosphere prior to observation. All samples were examined employing an accelerating voltage of 10 kV.

#### 2.4.11. Tensile Test

Tensile strength (TS) and elongation at break (EB) were determined using a 5967 Universal Testing Machine (Instron Systems, Barcelona, Spain), equipped with a tensile load cell of 250 N. According to ASTM D638-03 [25], the crosshead speed was set at 1 mm/min and samples with 22.25-mm length and 4.75-mm width were used.

#### 2.4.12. Co-Culturing of *Aspergillus nidulans* and Bacterial Strains

**Figure 1:**
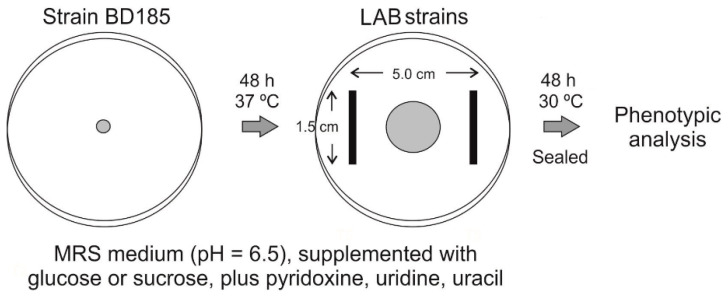
Procedure followed for the assessment of the ability of lactic acid bacteria (LAB) strains to inhibit active growth of *A. nidulans* strain BD185. Plates were analyzed in duplicates.

To assess the ability of LAB strains to inhibit active growth of *A. nidulans* strain BD185, the procedure described in Figure 1 was followed. Briefly, the fungal strain was point-inoculated on the center of Petri plates filled with the above described MRS media (see Section 2.2). After 48 h of culture at 37 °C, bacterial strains were inoculated on both sides of the fungal colony, maintaining the distances indicated in Figure 1. Plates were sealed with parafilm and cultured at 30 °C for additional 48 h before phenotypes were recorded.

**Figure 2:**
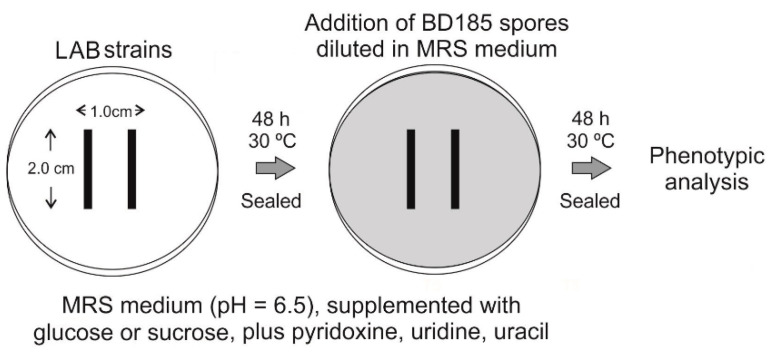
Procedure followed for the assessment of the ability of LAB strains to inhibit or delay germination of *A. nidulans* strain BD185. Plates were analyzed in duplicates.

To analyze the potential of LAB strains to inhibit or delay germination of asexual spores of *A. nidulans*, the procedure described in Figure 2 was followed [26,27]. Bacterial strains were inoculated in the center of MRS plates by drawing two lines of approximately 2 cm each at a relative distance of 1 cm. Plates were sealed and cultured at 30 °C for 48 h. Then, asexual spores of strain BD185 (6 × 10^5^ conidia, diluted in 8 mL of the same MRS medium as in the Petri plate) were added. Petri plates were sealed again and further incubated at 30 °C for 48 h before phenotypic analysis.

**Figure 3:**
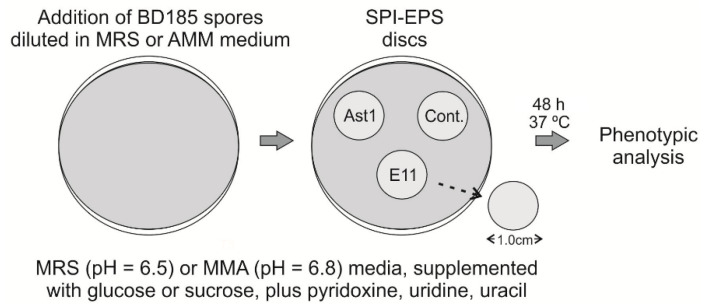
Procedure followed for the assessment of the effect of soy protein isolate (SPI)-exopolysaccharides (EPS) films on germination of asexual spores of strain BD185 of *A. nidulans*. Plates were analyzed in duplicates.

Finally, with the aim of assessing the potential of SPI-EPS films to inhibit germination of fungal spores, discs of a diameter of 1 cm were cut and sterilized with UV light. Conidia dilutions (6 × 10^5^ or 4 × 10^4^ conidia, respectively, diluted in 8 mL of the same MRS or AMM media as in the Petri plates) were poured on previously prepared MRS or AMM plates (Figure 3). After medium solidification, sterilized discs were located on the surface of the plates. Phenotypes were recorded after 48 h of culture at 37 °C.

#### 2.4.13. Statistical Analysis

Analysis of variance (ANOVA) was used to determine the significance of differences among samples. The analysis was performed with a SPSS computer program (SPSS Statistic 23.0, Barcelona, Spain) and Tukey’s test was used for multiple comparisons. At least three replicates were carried out. Differences were statistically significant at the *p* < 0.05 level.

## 3. Results and Discussion

### 3.1. Optical Properties

First of all, film appearance-related properties were analyzed. Hence, film color and gloss were determined and values are shown in Table 1. It is worth noting that all films showed high L* values, indicating the lightness of control and EPS-incorporated SPI films. Additionally, all films showed a yellowish color which is characteristic of SPI materials [28]. This yellowness, indicated by the positive values of b* parameter, was more intense for SPI-E11 films, while SPI-AST1 films showed a less intense yellowish color in comparison to control films. As a consequence, ΔE* parameter changed (*p* < 0.05), although this change was not relevant since it was lower than 5 and, thus, was non-perceptible to the human eye [29]. Furthermore, gloss values were measured and are shown in Table 1. As can be observed, all films presented low values of gloss, indicating non-glossy and rough surfaces [30], which became even rougher when EPS were incorporated into the formulation.

**Figure 4:**
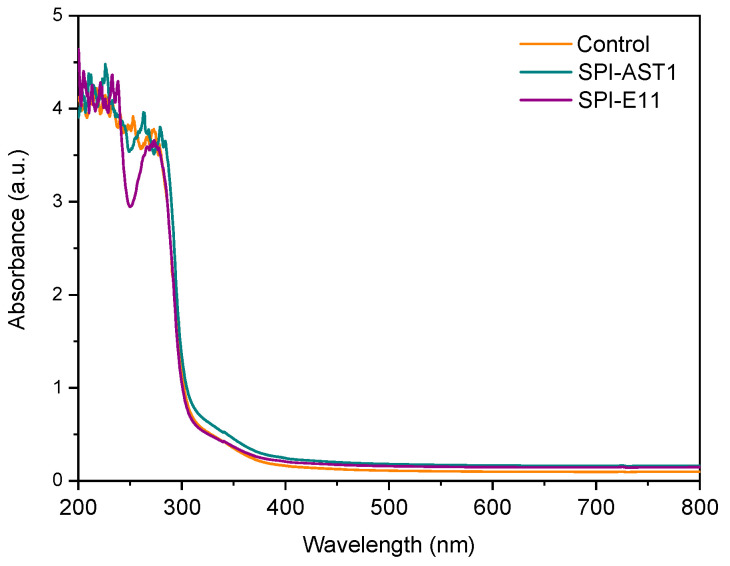
UV-Vis spectra of control and EPS-containing films.

Film transparency was measured by the absorbance at 600 nm in UV-Vis spectra. As can be seen in Figure 4, no absorbance was observed at this wavelength, indicating that films were transparent. Additionally, UV-Vis spectroscopy was carried out to determine light barrier properties of SPI films. As can be observed in Figure 4, SPI films provided an excellent UV barrier from 200 to 300 nm, due to the high percentage of aromatic amino acids (tyrosine, tryptophan and phenylalanine) present in soy protein [31,32]. Moreover, this UV barrier was improved in EPS-containing films probably due to the presence of hydroxyl groups in EPS, which have been described to act as auxochrome groups [33,34].

### 3.2. Thermal Properties of Films

**Figure 5:**
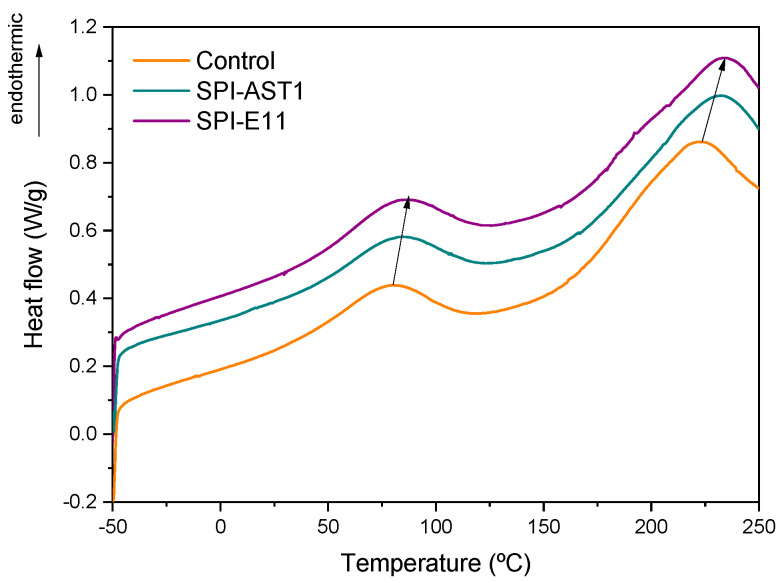
DSC thermograms of control and exopolysaccharide-containing films.

Thermal properties of SPI films were analyzed by DSC and TGA. Regarding DSC analysis (Figure 5), all films displayed an endothermic peak around 80–86 °C, associated to the denaturation of 7S protein fraction, the fraction of lower molecular weight. Another peak appeared around 220–235 °C, corresponding to the thermal denaturation of glycinin or 11S protein fraction, the fraction of higher molecular weight [35]. Denaturation temperatures are dependent on moisture content and shift to higher values at lower moisture contents [36]; therefore, these results would be indicative of lower moisture contents for the EPS-incorporating films.

**Figure 6:**
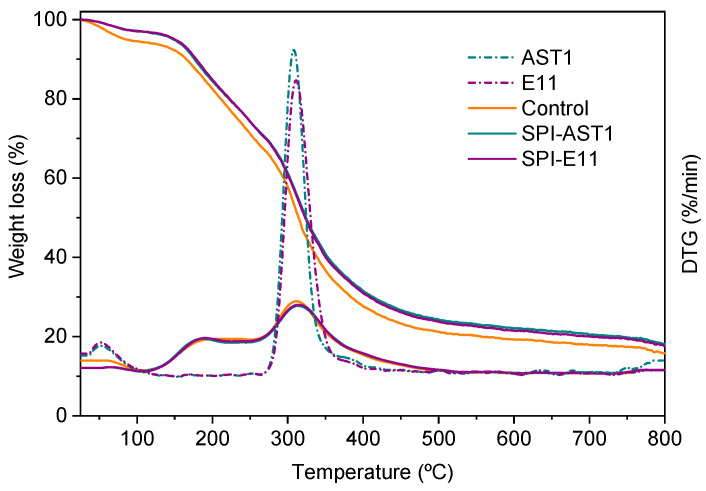
Weight loss of control and EPS-containing films, as well as DTG curves of control and EPS-containing films in comparison to DTG curves of pure EPS (dotted curves).

Concerning thermo-gravimetric analysis, all films showed three main weight loss steps (Figure 6). The initial weight loss around 100 °C was associated to the evaporation of moisture in the film. The second stage around 190 °C was related to the evaporation of glycerol [37]. Finally, the third stage around 312 °C was an overlapping decomposition region, in which the thermal breakage of backbone bonds among peptides in SPI chain, such as C–C, C–N, and C–O bonds, occurred [38,39]. This third stage also involves the decomposition of EPS, as can be observed in comparison to DTG curves of AST1 and E11, also shown in Figure 6.

### 3.3. Physicochemical Properties

**Figure 7:**
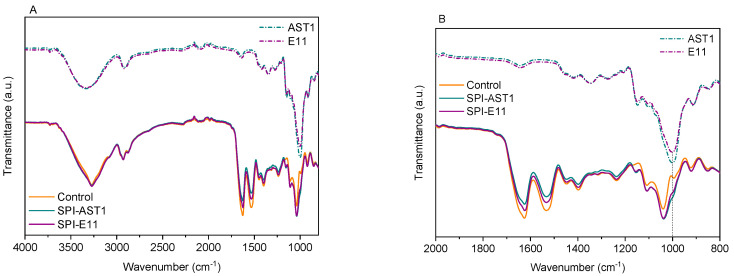
FTIR spectra of pure EPS (dotted curves) and those of control and EPS-containing films (continue curves) (**A**) from 4000 to 800 cm^−1^ and (**B**) from 2000 to 800 cm^−1^.

The interactions among the different components of films were studied employing FTIR analysis. As shown in Figure 7A, the bands of SPI are related to O–H stretching and N–H bending, around 3275 cm^−1^; C=O stretching (amide I), at 1625 cm^−1^; N–H bending (amide II), at 1533 cm^−1^; and C–N stretching (amide III), at 1236 cm^−1^ [40]. The main five absorption bands of glycerol were located from 800 to 1150 cm^−1^, corresponding to the vibrations of C–C and C–O linkages [41]. Both EPS had similar spectra and showed the typical absorption bands of polysaccharides, mainly the strong band located at 1000 cm^−1^ related to C–O bonds in EPS [20]. The abovementioned absorption bands arose on all films’ spectra. Nevertheless, it is worth noting that the intensity of amide I and amide II bands decreased in EPS-containing films, while the intensity of the band at 1040 cm^−1^ increased, as can be seen more clearly in Figure 7B. Additionally, the small band at 1000 cm^−1^ in SPI films became a shoulder in EPS-containing films. Since the appearance of new bands was not observed by FTIR analysis, it can be said that no chemical reaction occurred between SPI and EPS, and the changes observed in the intensity of the abovementioned bands can be related to physical interactions by hydrogen bonding between amino and hydroxyl groups of SPI and hydroxyl groups of EPS.

This physical crosslinking between SPI and EPS led to a reduction of moisture content in films [42,43]. Although no significant (*p* > 0.05) difference was observed for MC values of SPI-AST1 (17.12 ± 1.17%) and SPI-E11 17.97 ± 0.10%), these values were significantly (*p* < 0.05) lower than those of control films (20.58 ± 0.51%). This decrease in MC values can be explained by the interactions among the polar groups of SPI and EPS shown by FTIR, resulting in less available polar groups to interact with moisture. These results are in accordance with the increase in denaturation temperatures observed by DSC analysis (Figure 5).

**Figure 8:**
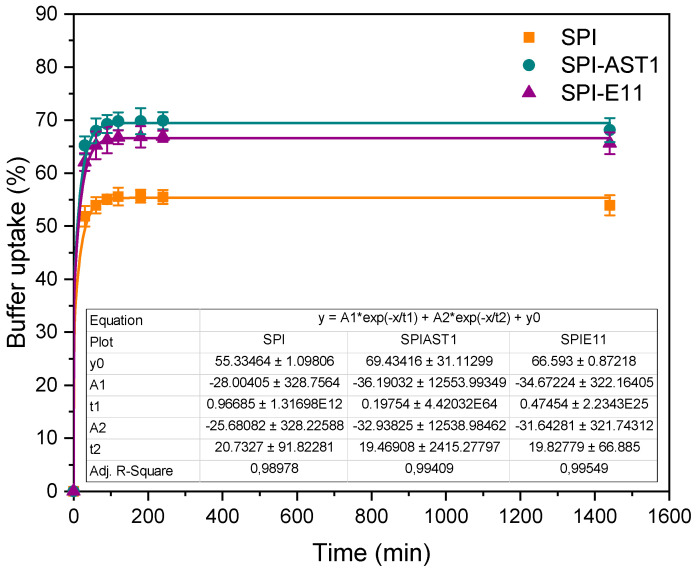
Buffer uptake of control and EPS-containing films.

For further assessment of the physical interactions effect in the physicochemical behavior of the films, buffer uptake was measured. As can be seen in Figure 8, all films swelled rapidly from the beginning of the test and BU values were maintained after one day. It is worth noting that the addition of EPS caused an increase in BU values. Since swelling is directly associated with cross-linking [44], these results can be related to the physical interactions formed between protein and EPS, which could lead to a looser structure, facilitating buffer uptake.

### 3.4. Morphological and Mechanical Properties

**Figure 9:**
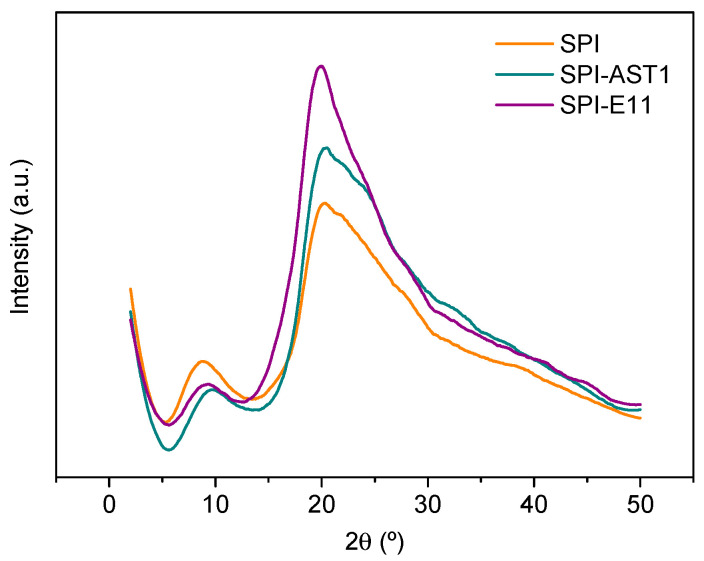
XRD patterns of control and EPS-containing films.

In order to relate the properties of the films to their structure, XRD and SEM analyses were carried out. As can be seen in Figure 9, two peaks appeared around 9° and 20°, associated to the α-helix and β-sheet structures of the soy protein secondary conformation, respectively [45]. Among the samples under study, when EPS were added, the most relevant difference was related to the increase in the intensity in the peak located around 20°, which indicated that the degree of structural order also increased [46], due to the new interactions formed between SPI and EPS, as shown in FTIR spectra.

**Figure 10:**
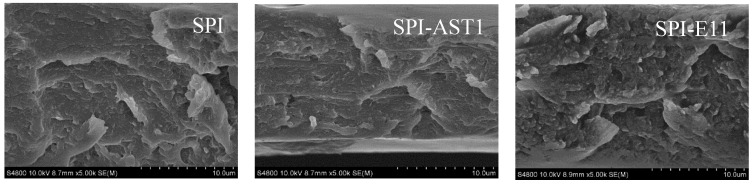
SEM micrographs of control and EPS-containing films.

Additionally, SEM analysis was carried out in order to observe the morphology of the film cross-section. As can be seen in Figure 10, all films presented homogeneous and rough structures and no relevant difference was appreciated among the cross-sections shown in SEM images, although SPI-E11 films seemed to offer the roughest cross-section. Therefore, it was concluded that EPS were well-distributed into the films at a microscopic level.

Finally, the mechanical properties of the films were evaluated by measuring the values of tensile strength (TS) and elongation at break (EB) (Table 2). There was no significant (*p* > 0.05) difference in TS values, in accordance with the similar cross-sections observed in SEM images (Figure 10). In contrast, EB values decreased (*p* < 0.05) with the incorporation of EPS, in agreement with a more ordered structure, as shown in XRD analysis (Figure 9). Nevertheless, it is worth mentioning that the TS values of the SPI films prepared in this work by compression molding are higher than those observed for SPI films with the same plasticizer content (30% glycerol) but prepared by solution casting [47]. This confirms the suitability of this method, not only for scaling-up production but also for the improvement of technological properties.

### 3.5. Germination Delay of the Filamentous Fungus Aspergillus nidulans in Co-Culture with LAB Strains

Specific LAB strains isolated from a variety of food matrices have been found to show antifungal/fungistatic activity against fungal pathogens [48]; therefore, the potential of the LAB strains from our collection, as well as those of the SPI films containing the EPS produced by them, to inhibit or delay filamentous fungal growth and/or germination were assessed in this work. To that end, co-cultures were carried out with the fungus *Aspergillus nidulans*, which is a widely used reference ascomycete in the study of secondary metabolism and fungal–bacterial interactions [49,50,51].

**Figure 11:**
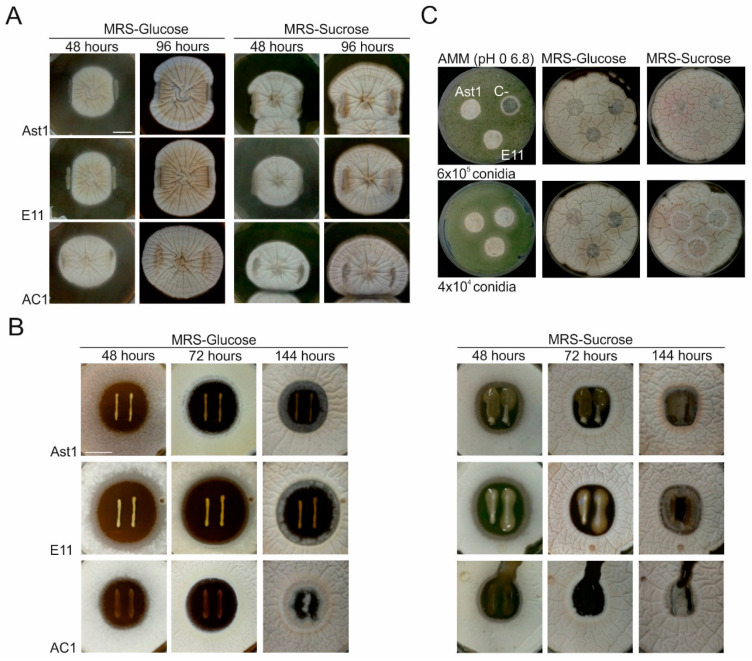
Fungal–bacterial co-cultures. (**A**) Assessment of the potential of LAB strains to inhibit or slow down active growth of hyphae of *A. nidulans*. Phenotypes were analyzed on MRS media (pH = 6.5) supplemented with glucose (**left**) or sucrose (**right**) after 48 h of culture at 37 °C plus additional 48 (column 1) and 96 h (column 2) of culture after inoculation of LAB strains AST1 (row 1), E11 (row 2) and AC1 (row 3) (see Figure 1). Scale bar = 2 cm. (**B**) Assessment of the potential of LAB strains to inhibit or delay germination of *A. nidulans* conidia. LAB strains (AST1, E11 and AC1 in rows 1, 2 and 3, respectively) were inoculated on the center of MRS plates supplemented with glucose (**left**) or sucrose (**right**). After 48 h of culture at 30 °C, a dilution of BD185 conidia was added to each plate (see Figure 2). Phenotypes were analyzed after 48, 72 or 96 h of culture at 30 °C. Scale bar = 2 cm. (**C**) Phenotype of *A. nidulans* growing on SPI-EPS films. Dilutions of 6 × 10^5^ (row 1) or 4 × 10^4^ (row 2) conidia in 8 mL of MRS or *Aspergillus* minimal medium (AMM) media were poured on previously prepared MRS or AMM plates (diameter: 9 cm) (see Figure 3). After addition of SPI-EPS films, plates were incubated for 48 h at 37 °C. C-: negative control, an SPI film without EPS.

First, the ability of LAB strains AC1 (used here as a reference), AST1 and E11 to inhibit active growth of *A. nidulans* strain BD185 was assessed (Figure 11A). Phenotypes were analyzed on MRS plates supplemented with glucose (Figure 11A, left), as a repressor of EPS production, or sucrose, as an inducer (Figure 11A, right) [20]. After 48 or 96 h of culture at 30 °C, the presence of LAB strains modified the growth pattern of the fungal strain (colonies use to be radial) in different ways. While LAB strains AST1 and E11 seemed to decrease radial extension of the fungal colony, strain AC1 seemed to stimulate it. The same behavior was observed in both MRS media used. Nevertheless, the fungus grew over the bacterial colonies faster in MRS medium supplemented with sucrose.

Second, we tested whether the same LAB strains as above could inhibit or delay germination of fungal asexual spores (also known as conidia). All LAB strains induced an inhibition zone in which germination of *A. nidulans* conidia was delayed to a variable extent (Figure 11B). E11 induced the biggest zone of inhibition (middle row in both panels in Figure 11B) and was the strain that best resisted fungal invasion compared to strains AST1 and AC1. This behavior was observed in both MRS media tested but, in general, the diameters of inhibition zones were bigger in a medium supplemented with glucose. In addition, invasion of inhibition zones by the fungus was faster in an MRS medium supplemented with sucrose, suggesting that this medium, and possibly the production of EPS, stimulates germination of fungal spores and growth of hyphae.

Finally, SPI-AST1 and SPI-E11 films were tested based on their potential to inhibit germination of *A. nidulans* conidia. Besides MRS media supplemented with glucose or sucrose, AMM, which is routinely adjusted to pH 6.8, was also used. A soy protein film which did not incorporate any EPS was used as a control. Results, mainly those obtained in AMM, showed that the presence of EPS did not cause an inhibition of spore germination, but seemed to slightly stimulate it.

Overall, results suggest that the inhibitory effect of our LAB strains on germination of fungal spores is not caused by EPS production and that culture conditions favoring EPS production stimulate filamentous fungal growth.

## 4. Conclusions

Two exopolysaccharides (EPS), produced by *Liquorilactobacillus* strains AST1 and E11, derived from vegetal sources, were incorporated into SPI film-forming formulations resulting in transparent and homogeneous films, suggesting the good compatibility between SPI and EPS, as observed by SEM images, which showed that EPS were well-distributed in the films. Additionally, the E11 strain showed potential antifungal/fungistatic activity against germination of *A. nidulans* spores. Furthermore, FTIR analysis indicated that new physical interactions were formed between SPI and EPS leading to films with a higher buffer uptake capacity, a property that can be relevant for pharmaceutical or biomedical applications.

## Figures and Tables

**Table 1:** Color and gloss values of control and EPS-containing films.

Film	L*	a*	b*	ΔE*	Gloss (GU)
Control SPI-AST1	94.56 ± 0.34 ^a^ 95.31 ± 0.45 ^b^	−0.84 ± 0.04 ^b^ −0.61 ± 0.03 ^a^	8.66 ± 0.31 ^b^ 6.78 ± 0.31 ^a^	--- 2.08 ± 0.29 ^a^	23.4 ± 2.7 ^a^ 18.1 ± 2.2 ^b^
SPI-E11	94.17 ± 0.20 ^a^	−0.91 ± 0.04 ^c^	11.93 ± 0.58 ^c^	3.29 ± 0.59 ^b^	20.9 ± 2.5 ^ab^

^a–c^ Two means followed by the same letter in the same column are not significantly (*p* > 0.05) different according to the Tukey’s multiple range test.

**Table 2:** Tensile strength (TS) and elongation at break (EB) values of control and EPS-containing films.

Film	E (MPa)	TS (MPa)	EB (%)
Control SPI-AST1	213.56 ± 16.68 ^a^ 278.59 ± 18.79 ^b^	12.47 ± 0.46 ^ab^ 13.03 ± 0.41 ^a^	48.17 ± 4.01 ^a^ 40.50 ± 5.41 ^b^
SPI-E11	342.51 ± 15.87 ^c^	11.54 ± 0.88 ^b^	25.04 ± 3.41 ^c^

^a–c^ Two means followed by the same letter in the same column are not significantly (*p* > 0.05) different according to the Tukey’s multiple range test.

## References

[1] Andrew M., Jayaraman G. (2020). Structural features of microbial exopolysaccharides in relation to their antioxidant activity. Carbohydr. Res..

[2] Hu X., Li D., Qiao Y., Wang X., Zhang Q., Zhao W., Huang L. (2020). Purification, characterization and anticancer activities of exopolysaccharide produced by *Rhodococcus erythropolis* HX-2. Int. J. Biol. Macromol..

[3] Schmid J. (2018). Recent insights in microbial exopolysaccharide biosynthesis and engineering strategies. Curr. Opin. Biotechnol..

[4] Food and Drug Administration (2018). Microorganisms & Microbial-Derived Ingredients Used in Food (Partial List). https://www.fda.gov/food/generally-recognized-safe-gras/microorganisms-microbial-derived-ingredients-used-food-partial-list.

[5] Oleksy-Sobczak M., Klewicka E., Piekarska-Radzik L. (2020). Exopolysaccharides production by *Lactobacillus rhamnosus* strains—Optimization of synthesis and extraction conditions. LWT-Food Sci. Technol..

[6] Hu Y., Gänzle M.G. (2018). Effect of temperature on production of oligosaccharides and dextran by *Weissella cibaria* 10 M. Int. J. Food Microbiol..

[7] Lakshmi Bhavani A., Nisha J. (2010). Dextran - the polysaccharide with versatile uses. Int. J. Pharma Bio Sci..

[8] Lynch K.M., Coffey A., Arendt E.K. (2018). Exopolysaccharide producing lactic acid bacteria: Their techno-functional role and potential application in gluten-free bread products. Food Res. Int..

[9] Saravanan C., Shetty P.K.H. (2016). Isolation and characterization of exopolysaccharide from *Leuconostoc lactis* KC117496 isolated from idli batter. Int. J. Biol. Macromol..

[10] Zarour K., Llamas M.G., Prieto A., Ruas-Madiedo P., Dueñas M.T., de Palencia P.F., Aznar R., Kihal M., Lopez P. (2017). Rheology and bioactivity of high molecular weight dextrans synthesised by lactic acid bacteria. Carbohydr. Polym..

[11] Mahgoub A.M., Mahmoud M.G., Selim M.S., Awady M.E.E. (2018). Exopolysaccharide from marine *Bacillus velezensis* MHM3 induces apoptosis of human breast cancer MCF-7 cells through a mitochondrial pathway. Asian Pac. J. Cancer Prev..

[12] Raposo M.F.J., de Morais R.M.S.C., de Morais A.M.M.B. (2013). Bioactivity and applications of sulphated polysaccharides from marine microalgae. Mar. Drugs.

[13] Hussain A., Zia K.M., Tabasum S., Noreen A., Ali M., Iqbal R., Zuber M. (2017). Blends and composites of exopolysaccharides; properties and applications: A review. Int. J. Biol. Macromol..

[14] Etxabide A., Garrido T., Uranga J., Guerrero P., de la Caba K. (2018). Extraction and incorporation of bioactives into protein formulations for food and biomedical applications. Int. J. Biol. Macromol..

[15] Garrido T., Leceta I., de la Caba K., Guerrero P. (2018). Chicken feathers as a natural source of sulphur to develop sustainable protein films with enhanced properties. Int. J. Biol. Macromol..

[16] Wang S., Marcone M., Barbut S., Lim L.T. (2012). The impact of anthocyanin-rich red raspberry extract (ARRE) on the properties of edible soy protein isolate (SPI) films. J. Food Sci..

[17] Zou Y.C., Wu C.L., Ma C.F., He S., Brennan C.S., Yuan Y. (2019). Interactions of grape seed procyanidins with soy protein isolate: Contributing antioxidant and stability properties. LWT-Food Sci. Technol..

[18] Galus S. (2018). Functional properties of soy protein isolate edible films as affected by rapeseed oil concentration. Food Hydrocoll..

[19] De Man J.C., Rogosa M., Sharpe M.E. (1960). A medium for the cultivation of lactobacilli. J. Appl. Bacteriol..

[20] Llamas-Arriba M.G., Puertas A.I., Prieto A., López P., Cobos M., Miranda J.I., Marieta C., Ruas-Madiedo P., Dueñas M.T. (2019). Characterization of dextrans produced by *Lactobacillus mali* CUPV271 and *Leuconostoc carnosum* CUPV411. Food Hydrocoll..

[21] Herrero-Garcia E., Perez-de Nanclares-Arregi E., Cortese M.S., Markina-Iñarrairaegui A., Oiartzabal-Arano E., Etxebeste O., Ugalde U., Espeso E.A. (2015). Tip-to-nucleus migration dynamics of the asexual development regulator FlbB in vegetative cells. Mol. Microbiol..

[22] Otamendi A., Perez-de Nanclares-Arregi E., Oiartzabal-Arano E., Cortese M.S., Espeso E.A., Etxebeste O. (2019). Developmental regulators FlbE/D orchestrate the polarity site-to-nucleus dynamics of the fungal bZIP transcription factor FlbB. Cell. Mol. Life Sci..

[23] Etxebeste O., Ni M., Garzia A., Kwon N.J., Fischer R., Yu J.H., Espeso E.A., Ugalde U. (2008). Basic-zipper-type transcription factor FlbB controls asexual development in *Aspergillus nidulans*. Eukaryot. Cell.

[24] ASTM D523-14 (2014). Standard test method for specular gloss. Annual Book of ASTM Standards.

[25] ASTM D638-03 (2003). Standard test method for tensile properties of plastics. Annual Book of ASTM Standards.

[26] Bartkiene E., Lele V., Ruzauskas M., Domig K.J., Starkute V., Zavistanaviciute P., Bartkevics V., Pugajeva I., Klupsaite D., Juodeikiene G. (2020). Lactic acid bacteria isolation from spontaneous sourdough and their characterization including antimicrobial and antifungal properties evaluation. Microorganisms.

[27] Magnusson J., Schnürer J. (2001). *Lactobacillus coryniformis* subsp. *coryniformis* strain Si3 produces a broad-spectrum proteinaceous antifungal compound. Appl. Environ. Microbiol..

[28] Garrido T., Etxabide A., Peñalba M., de la Caba K., Guerrero P. (2013). Preparation and characterization of soy protein thin films: Processing-properties correlation. Mater. Lett..

[29] Luchese C.L., Abdalla V.F., Spada J.C., Tessaro I.C. (2018). Evaluation of blueberry residue incorporated cassava starch film as pH indicator in different simulants and foodstuffs. Food Hydrocoll..

[30] Acosta S., Jiménez A., Cháfer M., González-Martínez C., Chiralt A. (2015). Physical properties and stability of starch-gelatin based films as affected by the addition of esters of fatty acids. Food Hydrocoll..

[31] Ren C., Xiong W., Peng D., He Y., Zhou P., Li J., Li B. (2018). Effects of thermal sterilization on soy protein isolate/polyphenol complexes: Aspects of structure, in vitro digestibility and antioxidant activity. Food Res. Int..

[32] Wang Y., Zhang A., Wang X., Xu N., Jiang L. (2020). The radiation assisted-Maillard reaction comprehensively improves the freeze-thaw stability of soy protein-stabilized oil-in-water emulsions. Food Hydrocoll..

[33] Sudha, Gupta C., Aggarwal S. (2016). Dyeing wet blue goat nappa skin with a microbial colorant obtained from *Penicillium minioluteum*. J. Clean. Prod..

[34] Zhou B., Gao M., Feng X., Huang L., Huang Q., Kootala S., Larsson T.E., Zheng L., Bowden T. (2020). Carbazate modified dextrans as scavengers for carbonylated proteins. Carbohydr. Polym..

[35] Mo X., Sun X. (2002). Plasticization of soy protein polymer by polyol-based plasticizers. J. Am. Oil Chem. Soc..

[36] Garrido T., Leceta I., Cabezudo S., Guerrero P., de la Caba K. (2016). Tailoring soy protein film properties by selecting casting or compression as processing methods. Eur. Polym. J..

[37] Garrido T., Uranga J., Guerrero P., de la Caba K., Gutierrez T.J. (2018). The potential of vegetal and animal proteins to develop more sustainable food packaging. Polymers for Food Applications.

[38] Kang H., Wang Z., Zhang W., Li J., Zhang S. (2016). Physico-chemical properties improvement of soy protein isolate films through caffeic acid incorporation and tri-functional aziridine hybridization. Food Hydrocoll..

[39] Zheng T., Yu X., Pilla S. (2017). Mechanical and moisture sensitivity of fully bio-based dialdehyde carboxymethyl cellulose cross-linked soy protein isolate films. Carbohydr. Polym..

[40] Han Y., Yu M., Wang L. (2018). Preparation and characterization of antioxidant soy protein isolate films incorporating licorice residue extract. Food Hydrocoll..

[41] Uranga J., Etxabide A., Cabezudo S., de la Caba K., Guerrero P. (2020). Valorization of marine-derived biowaste to develop chitin/fish gelatin products as bioactive carriers and moisture scavengers. Sci. Total Environ..

[42] Carpiné D., Dagostin J.L.A., Bertan L.C., Mafra M.R. (2015). Development and characterization of soy protein isolate emulsion-based edible films with added coconut oil for olive oil packaging: Barrier, mechanical, and thermal properties. Food Bioprocess Technol..

[43] Qu P., Huang H., Wu G., Sun E., Chang Z. (2015). Effects of hydrolysis degree of soy protein isolate on the structure and performance of hydrolyzed soy protein isolate/urea/formaldehyde copolymer resin. J. Appl. Polym. Sci..

[44] Desam G.P., Li J., Chen G., Campanella O., Narsimhan G. (2018). Prediction of swelling behavior of crosslinked maize starch suspensions. Carbohydr. Polym..

[45] Ye Q., Han Y., Zhang J., Zhang W., Xia C., Li J. (2019). Bio-based films with improved water resistance derived from soy protein isolate and stearic acid *via* bioconjugation. J. Clean. Prod..

[46] Garrido T., Etxabide A., Leceta I., Cabezudo S., de la Caba K., Guerrero P. (2014). Valorization of soya by-products for sustainable packaging. J. Clean. Prod..

[47] Guerrero P., Retegi A., Gabilondo N., de la Caba K. (2010). Mechanical and thermal properties of soy protein films processed by casting and compression. J. Food Eng..

[48] Sadiq F.A., Yan B., Tian F., Zhao J., Zhang H., Chen W. (2019). Lactic acid bacteria as antifungal and anti-mycotoxigenic agents: A comprehensive review. Compr. Rev. Food Sci. Food Saf..

[49] Etxebeste O., Espeso E.A. (2020). *Aspergillus nidulans* in the post-genomic era: A top-model filamentous fungus for the study of signaling and homeostasis mechanisms. Int. Microbiol..

[50] Nützmann H.W., Scazzocchio C., Osbourn A. (2018). Metabolic gene clusters in eukaryotes. Annu. Rev. Genet..

[51] Schroeckh V., Scherlach K., Nützmann H.W., Shelest E., Schmidt-Heck W., Schuemann J., Martin K., Hertweck C., Brakhage A.A. (2009). Intimate bacterial-fungal interaction triggers biosynthesis of archetypal polyketides in *Aspergillus nidulans*. Proc. Natl. Acad. Sci. USA.

